# The esBAF and ISWI nucleosome remodeling complexes influence occupancy of overlapping dinucleosomes and fragile nucleosomes in murine embryonic stem cells

**DOI:** 10.1186/s12864-023-09287-4

**Published:** 2023-04-13

**Authors:** David C. Klein, Kris Troy, Sarah A. Tripplehorn, Sarah J. Hainer

**Affiliations:** 1 Department of Biological Sciences, University of Pittsburgh, Pittsburgh, PA 15213 USA; 2 Department of Quantitative and Systems Biology, University of California, 95343 Merced, Merced, CA USA

**Keywords:** Nucleosomes, Chromatin, Remodeling, Stem cells, Subnucleosomes, Overlapping dinucleosomes, Fragile nucleosomes

## Abstract

**Background:**

Nucleosome remodeling factors regulate the occupancy and positioning of nucleosomes genome-wide through ATP-driven DNA translocation. While many nucleosomes are consistently well-positioned, some nucleosomes and alternative nucleosome structures are more sensitive to nuclease digestion or are transitory. Fragile nucleosomes are nucleosome structures that are sensitive to nuclease digestion and may be composed of either six or eight histone proteins, making these either hexasomes or octasomes. Overlapping dinucleosomes are composed of two merged nucleosomes, lacking one H2A:H2B dimer, creating a 14-mer wrapped by ~ 250 bp of DNA. In vitro studies of nucleosome remodeling suggest that the collision of adjacent nucleosomes by sliding stimulates formation of overlapping dinucleosomes.

**Results:**

To better understand how nucleosome remodeling factors regulate alternative nucleosome structures, we depleted murine embryonic stem cells of the transcripts encoding remodeler ATPases BRG1 or SNF2H, then performed MNase-seq. We used high- and low-MNase digestion to assess the effects of nucleosome remodeling factors on nuclease-sensitive or “fragile” nucleosome occupancy. In parallel we gel-extracted MNase-digested fragments to enrich for overlapping dinucleosomes. We recapitulate prior identification of fragile nucleosomes and overlapping dinucleosomes near transcription start sites, and identify enrichment of these features around gene-distal DNaseI hypersensitive sites, CTCF binding sites, and pluripotency factor binding sites. We find that BRG1 stimulates occupancy of fragile nucleosomes but restricts occupancy of overlapping dinucleosomes.

**Conclusions:**

Overlapping dinucleosomes and fragile nucleosomes are prevalent within the ES cell genome, occurring at hotspots of gene regulation beyond their characterized existence at promoters. Although neither structure is fully dependent on either nucleosome remodeling factor, both fragile nucleosomes and overlapping dinucleosomes are affected by knockdown of BRG1, suggesting a role for the complex in creating or removing these structures.

**Supplementary Information:**

The online version contains supplementary material available at 10.1186/s12864-023-09287-4.

## Background

The eukaryotic genome is packaged into chromatin, which is composed of structural and regulatory proteins, DNA, and RNA and condenses into higher-order structures that regulate DNA access by DNA binding factors [1, 2]. The primary structural protein components of chromatin, histones, act as packaging elements when assembled as a multimer with ~ 147 base pairs (bp) of DNA, which is termed the nucleosome [3]. A canonical nucleosome contains an octamer composed of two H2A:H2B dimers and a single H3:H4 tetramer, but nucleosome diversity can impart distinct identities necessary for regulatory function, and precise nucleosome compositions are often found at genomic regions with specific epigenetic and transcriptional roles [1, 2, 4, 5]. Nucleosome diversity can be generated when canonical histone proteins are replaced with structurally distinct histone variants—such as H3.3, H2A.Z, and CENP-A.

Nucleosome diversity can also come from the number of histone proteins assembled—nucleosomes with fewer than eight histones that are wrapped by fewer base pairs of DNA than traditional nucleosomes are called subnucleosomes [6–11]. Hexasomes, a type of subnucleosome, are formed when an H2A:H2B dimer is removed from a canonical nucleosome during transcription elongation or DNA replication and/or repair [10]. A hexasome wraps ~100–110 bp of DNA with an H2A:H2B dimer asymmetrically detached at the entry or exit site [10]. Similarly, tetrasomes, containing only the H3:H4 tetramer wrapped by ~80 bp of DNA, are formed during DNA-based events such as replication and transcription [11–13]. Although the full genomic circumstances under which subnucleosomes are created have not been fully clarified, some patterns in their localization have been observed. For example, in *S. cerevisiae*, hexasomes or tetrasomes have been observed at “wide” nucleosome depleted regions (NDRs), which are generally promoters of highly-transcribed housekeeping genes, and have been described as “fragile nucleosomes”, as they are observed in only light nuclease treatment conditions [14–16]. Fragile nucleosomes have not been extensively studied but are known to occupy CTCF binding sites, in addition to promoter regions [14–17].

Another alternative nucleosome structure is the “overlapping dinucleosome” (hereafter referred to as “OLDN”). OLDNs are formed through the collision of two adjacent nucleosomes, shown in vitro to be stimulated by the remodeling action of some nucleosome remodeling complexes [18–20]. This collision event leads to the ejection of one H2A:H2B dimer, forming a chimeric 14-mer composed of one hexasome and one octasome [18]. A crystal structure of OLDNs revealed that ~ 250 bp of DNA encircle the composite OLDN with one acidic patch shielded on the octasome, while another is missing entirely from the hexasome [21]. Nucleosome acidic patches are binding sites for nucleosome binding proteins, and binding of one such protein (RCCt) was markedly reduced after OLDN formation [21]. Additional changes between histone-DNA interactions at specific residues may compact and lend stability to the OLDN structure. While most OLDN studies have been performed in vitro, evidence of the existence of presumptive OLDNs in vivo was shown using HeLa cells [21].

The physical presence of nucleosomes is generally considered a barrier to binding of DNA-binding proteins, and the presence of a stably positioned nucleosome inhibits transcription [2, 22]. The location and composition of nucleosomes (and variants of nucleosomes) are therefore carefully regulated by nucleosome remodelers, which move or eject nucleosomes, or moderate histone variant exchange by utilizing ATP-hydrolysis to translocate DNA [23]. The protein domain content of the ATPase subunit classifies nucleosome remodelers into four subfamilies—CHD, INO80, ISWI, and SWI/SNF. These subfamily classifications are based primarily on structural characteristics; the function and mechanism of action for a given remodeling factor may differ from another even for ATPases found within the same family.

SWI/SNF complexes (termed mSWI/SNF or BAF [Brahma-Associated Factor] complexes in mammals) canonically facilitate chromatin accessibility through nucleosome eviction or catalytic translocation of DNA along the nucleosome array (sliding) [4, 23, 24]. SWI/SNF is required for activating transcription of many genes through maintaining appropriate occupancy of NDRs, but most subunits of SWI/SNF are not essential for yeast viability [25–27]. BAF complexes predominantly maintain chromatin accessibility at cis-regulatory elements (promoters and enhancers), but also repress pervasive transcription from non-coding DNA elements [28–30]. Closely related to SWI/SNF, the RSC complex was originally identified in *S. cerevisiae* based in large part on to its homology to the SWI/SNF complex [31]. Like SWI/SNF, RSC maintains NDRs between − 1 and + 1-positioned nucleosomes at promoters to regulate gene transcription [31–33, 44, 65]. Unlike SWI/SNF, however, most RSC subunits are essential in budding yeast, and RSC is much more abundant than SWI/SNF [31].

Interestingly, yeast RSC can produce alternative nucleosome structures through in vitro nucleosome sliding assays and in vivo profiling, including fragile nucleosomes, subnucleosomes, and OLDNs, while mammalian BAF complexes have been shown to produce subnucleosomes [7, 9, 15, 18, 19]. The conserved sliding action used by both RSC and BAF promotes the collision of adjacent nucleosomes, and may therefore drive the formation of fragile nucleosomes and/or OLDNs in vivo, in agreement with in vitro nucleosome sliding assays [18].

SWI/SNF remodelers are not the only nucleosome remodelers that act on alternative nucleosomes. Recently, yeast INO80 complex was shown to prefer a hexasome substrate to a full nucleosome for remodeling, where both in vitro and in vivo studies support a mechanism for rapid repositioning of hexasomes to prevent aberrant transcription within gene bodies [34]. Interestingly, INO80 is dependent on the nucleosome acidic patch for sliding activity, as are other nucleosome remodelers, including the ISWI nucleosome remodeler SNF2H [79]; however, these remodelers work with the acidic patch through distinct mechanisms. A recent cryo-EM structure of SNF2H bound to the nucleosome suggests a role for SNF2H in octamer deformation to stimulate directional nucleosome sliding [79].

Since little is known about the location and potential function of OLDNs and fragile nucleosomes in mammalian cells in vivo, we assayed alternative nucleosome localization and regulation in murine embryonic stem (ES) cells. To this end, we utilized micrococcal nuclease digestion coupled with deep sequencing (MNase-seq) to probe for the presence and genomic localization of OLDNs and fragile nucleosomes in ES cells. Because fragile nucleosomes are more sensitive to nuclease digestion than canonical nucleosomes [7, 14–17], we performed MNase-seq under both high- and low-MNase digestion conditions. In parallel, we paired MNase-seq with gel-extraction to enrich for ~ 250 bp DNA fragments, expected to be protected from MNase digestion by OLDNs. We examined differences in putative OLDN and fragile nucleosome occupancy and positioning in cells depleted of the ATPase subunit for esBAF (BRG1, encoded by the gene *Smarca4)* or ISWI1 (SNF2H, encoded by the gene *Smarca5*). Using these methods, we recapitulate identified fragile nucleosome and OLDN occupancy over TSS-proximal regions. Further, we identify BRG1-dependent fragile nucleosome occupancy at gene-distal DNase hypersensitive sites (DHSs) and BRG1-bound locations, as well as OLDN occupancy at gene-distal DHSs, CTCF binding sites, and locations bound by either BRG1 or SNF2H. We find that BRG1 depletion results in a reduction of fragile nucleosome occupancy, but an increase in OLDN occupancy over the same genomic regions. We speculate that this shift in occupancy signifies a role for the esBAF complex in breaking OLDNs into their hexasome and octasome components, leaving behind a canonical nucleosome and a fragile subnucleosome that is more susceptible to MNase digestion.

## Results

### Overlapping dinucleosomes are enriched at gene regulatory elements

**Fig. 1:**
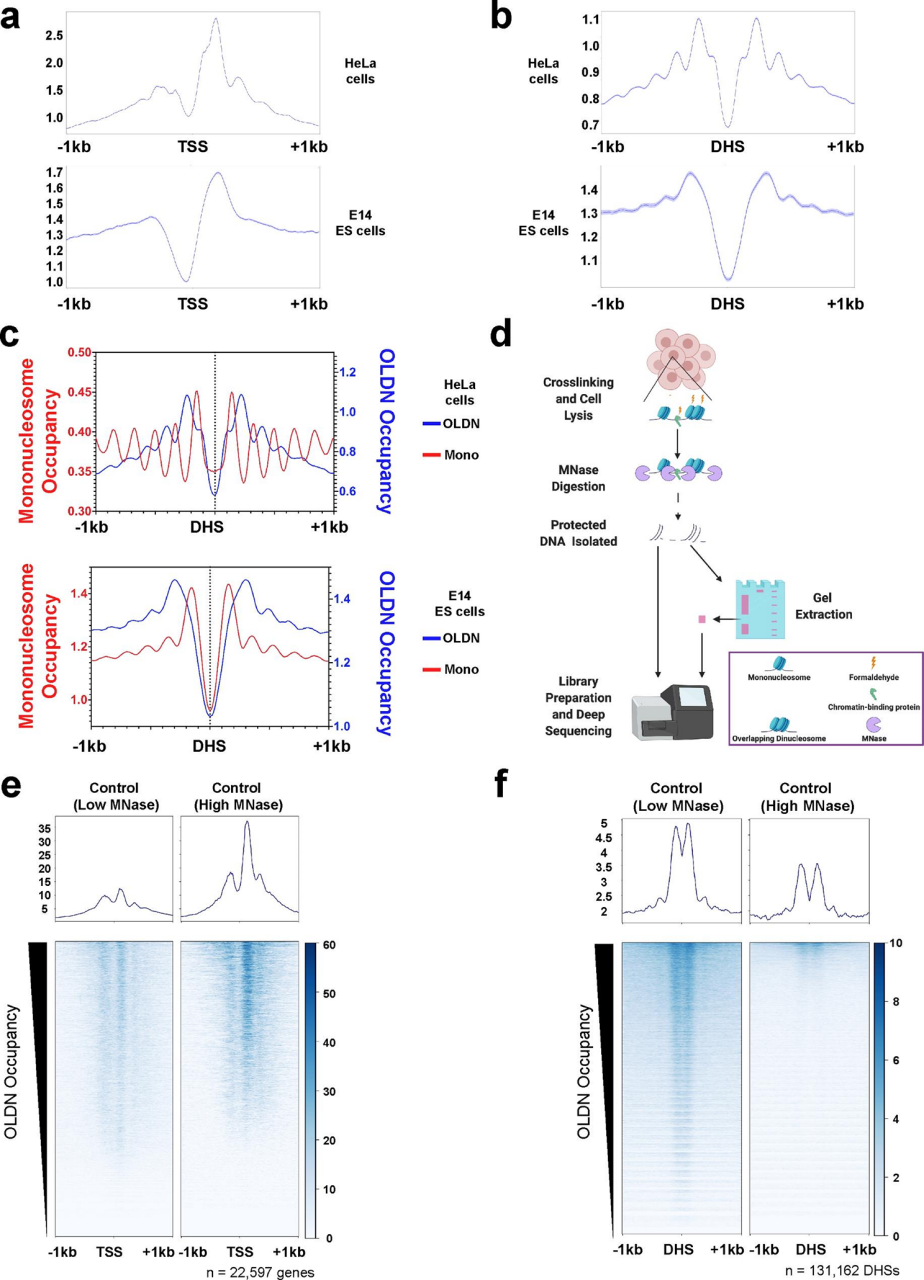
**Overlapping dinucleosomes are enriched at gene regulatory elements. (a)** Metaplots depicting average OLDN occupancy over all gene TSSs in human HeLa cells (top, data from DRP003456 [21]) and murine ES cells (bottom, data from GSE183278 [36]). Each line represents the mean of two biological replicates. OLDNs were identified bioinformatically using a fragment size selection between 230–270 bp. HeLa cell data were size-selected by gel extraction between 200–300 bp, while murine ES cell data were size-selected bioinformatically. **(b)** OLDN occupancy over gene-distal DNaseI hypersensitive sites (DHSs, GSM1014154 [37]) as in (a). n = 2 (mean). Data were processed as in panel a. **(c)** Overlaid OLDN and mononucleosome metaplots depicting spatial relationships between nucleosome structures at gene-distal DHSs. Fragment size was used to distinguish mononucleosomes (red, 135–165 bp) and OLDNs (blue, 230–270 bp). n = 2 (mean). Data from HeLa cells (top) and ES cells (bottom), processed as in panel a. **(d)** Schematic of MNase-seq experimental workflow and OLDN enrichment process, where parallel libraries were generated from the same sample: one including all MNase-digested products (unextracted) and one enriched for OLDNs (gel-extracted to enrich for fragments between ~ 200–300 bp). **e-f**. Metaplots and heatmaps depicting OLDN occupancy over all genes (e) and gene-distal DHSs (f) under conditions of low (left) and high (right) MNase digestion (DHSs from GSM1014154 [37]). OLDNs were enriched by gel extraction (~ 200–300 bp) and bioinformatically filtered by fragment size (230–270 bp). n = 3 (mean).

To determine OLDN localization across mammalian genomes we bioinformatically separated OLDN-sized DNA fragments (230–270 bp) from two published MNase-seq datasets and visualized occupancy at transcription start sites (TSSs), where OLDNs have been shown to occupy in HeLa cells (Fig. 1a) [36]. As expected, we were able to recapitulate the trends shown in HeLa cells, and consistently, we identified OLDNs near TSSs in murine embryonic stem (ES) cells (Fig. 1a, bottom) [21]. We hypothesized that OLDNs may be enriched at gene-distal regulatory elements, as these regions are hotspots for binding of regulatory factors, with high nucleosome turnover. Indeed, we identified strong enrichment of OLDNs flanking gene-distal DNaseI hypersensitive sites (DHSs) in both HeLa and murine ES cells (Fig. 1b), consistent with a model wherein OLDNs are generated through collisions with downstream nucleosomes in the process of clearing the DHS. In further support of this model, putative OLDNs are formed directly between adjacent mononucleosomes in both cell types (Fig. 1c).

Having bioinformatically identified OLDNs adjacent to TSSs and at gene-distal regulatory sites using available datasets, we performed modified MNase-seq experiments to enrich for putative OLDNs in murine ES cells (Fig. 1d). Briefly, we separated DNA protected following MNase-digestion into two parallel library preparations: one unextracted library, where we did not gel-extract to enrich for any size fragments, but rather constructed a library from the entire pool of protected DNA, and one using only gel-extracted DNA between ~200–300 bp as input for the library build (thus enriching for putative OLDNs). We validated our bioinformatic size selection by comparing a traditional mononucleosome size class (135–165 bp) with three putative OLDN-containing size classes (200–300 bp, 230–270 bp, and 245–255 bp) in our control (*EGFP* knockdown) MNase-seq dataset, as well as a published MNase-seq dataset (Fig. S1, GSE183278) [36]. We found that the all three putative OLDN-size classes resulted in similar profiles that were distinct from the mononucleosome size class (Fig. S1). Therefore, to allow for sufficient reads but also be as stringent as possible, we utilized 230–270 bp for the OLDN size class throughout our downstream analyses. We verified our MNase digestions by visual comparison on agarose gels (Fig. S2a).

To validate our gel extraction process, we plotted read counts as a histogram of fragment sizes, confirming that extracted libraries contained mostly reads between 200 and 300 bp whereas unextracted libraries had traditional MNase-digested fragment size profiles (Fig. S2b-m). To examine whether these newly generated datasets recapitulate known OLDN occupancy over TSSs and occupancy over gene-distal DHSs that we identified in public datasets, we visualized the OLDN-enriched MNase-seq data over these genomic locations (Fig. 1e,f). We found that in both low and high MNase digestion conditions, OLDNs are enriched at both TSSs and gene-distal DHSs. Together, our analysis of available datasets and newly generated data demonstrate OLDN localization to promoter and gene-distal regulatory elements.

### Overlapping dinucleosome enrichment correlates with transcription

**Fig. 2:**
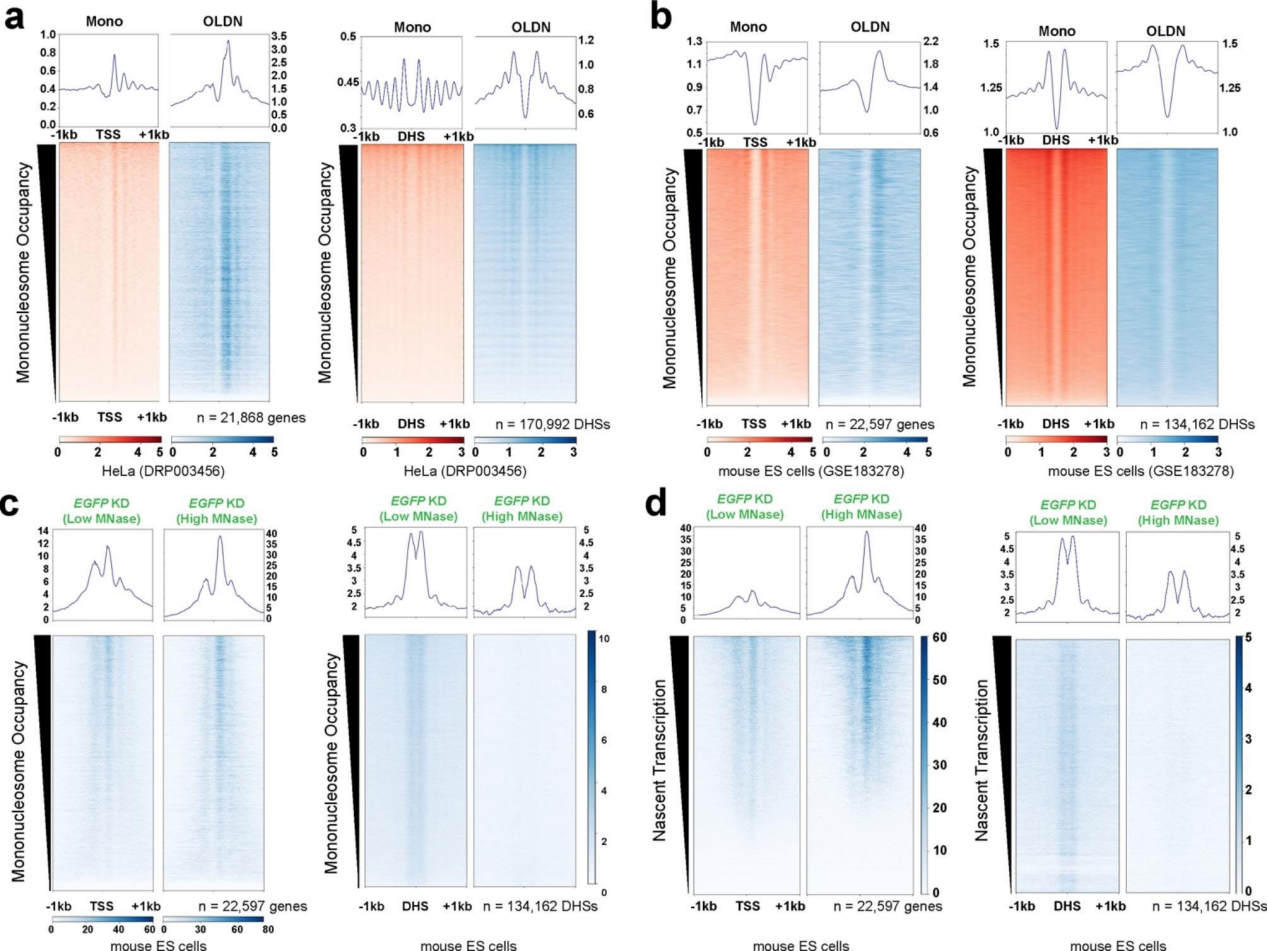
**OLDN occupancy qualitatively correlates with transcription, mononucleosome occupancy, and RNAPII binding. (a)** Mononucleosomes and OLDNs from HeLa cells (DRP003456 [21]), sorted by mononucleosome occupancy and visualized over hg38 RefSeq Select mRNA TSSs. Mononucleosome libraries were not gel-extracted and size-selected for fragments between 135–165 bp. OLDN libraries were gel-extracted and size-selected for fragments between 230–270 bp. n = 2. **(b)** As in panel a, but using mouse MNase-seq data (GSE183278 [36]) and visualized over mm10 RefSeq Select mRNA TSSs. Libraries were not gel-extracted but were bioinformatically size-selected for fragments between 135–165 bp (mononucleosomes) and 230–270 bp (OLDNs). n = 2. **(c)** Putative OLDNs identified from our EGFP KD (control) MNase-seq experiments visualized over TSSs (left) and gene-distal DHSs (right), sorted by mononucleosome occupancy. n = 3 merged replicates, shown as a single heatmap per condition. Gel-extracted libraries were used for these analyses and size-selected for fragments between 230–270 bp. **(d)** As in panel c, but sorted by nascent transcription (TT-seq data from GSE181624 [38]). Gel-extracted libraries were used for these analyses and size-selected for fragments between 230–270 bp.

Existing models for OLDN formation suggest they are formed through collision of two nucleosomes. Therefore, we anticipate OLDN occupancy to correlate with mononucleosome enrichment. To test this, we sorted our OLDN-enriched MNase-seq data and the previously published HeLa and ES cell datasets by mononucleosome occupancy from our paired, unextracted MNase-seq samples (bioinformatically selected for fragments between 135-165 bp) over TSSs and DHSs (Fig. 2a-c). We found an expected trend, where intensity of OLDN occupancy overall mimics mononucleosome occupancy over TSSs (Fig. 2a-c; left plots), but over gene-distal DHSs, there was a slightly less clear trend (Fig. 2a-c; right plots).

To identify a possible link between transcription and OLDN formation, we sorted the MNase-seq datasets by nascent transcription from the same cell line over both TSSs and gene-distal DHSs (Fig. 2d; TT-seq data from GSE181624 [38]). We observe a trend where OLDN occupancy qualitatively correlates with transcription, especially at TSSs. Together, our analyses support previously identified OLDN occupancy between the + 1/+2 nucleosomes near TSSs, but also identify non-genic, gene-distal DHSs where OLDNs are formed in HeLa and murine ES cells. While OLDNs occur at regions marked by transcription and mononucleosomes, both of which are likely necessary for OLDN formation, additional factors, such as nucleosome remodelers, may be important in generating OLDNs, in line with in vitro experiments [18, 21, 39].

### esBAF restricts OLDN occupancy

**Fig. 3:**
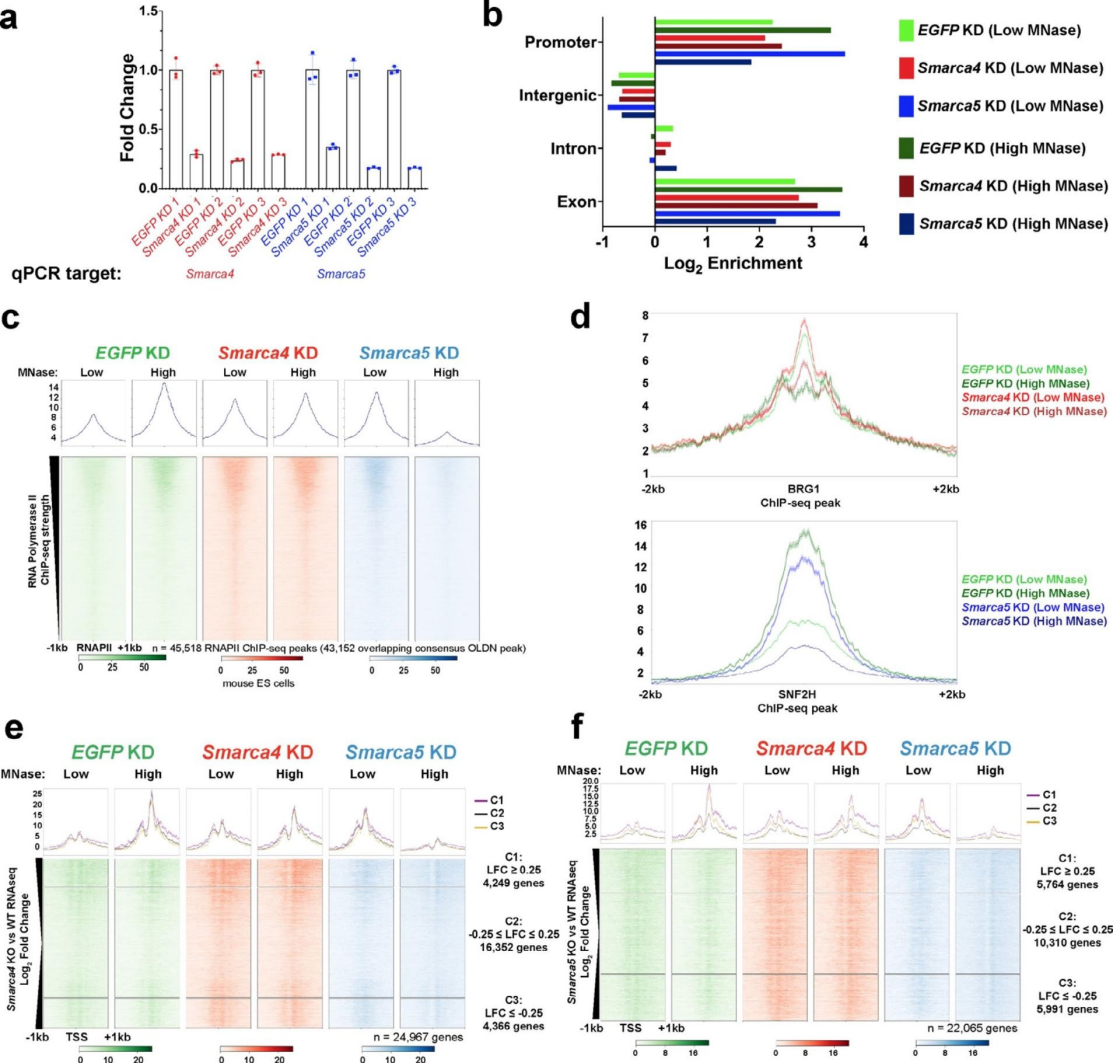
**Overlapping dinucleosome occupancy is dependent on the nucleosome remodeler BRG1. (a)** RT-qPCR results of three independent esiRNA knockdowns targeting *EGFP* (unexpressed control), the esBAF ATPase BRG1 (encoded by *Smarca4*) and the ISWI ATPase SNF2H (encoded by *Smarca5*). qPCR primers amplifying *Smarca4* (blue) and *Smarca5* (red) were used, and all values were normalized to *Gapdh* mRNA levels using ∆∆Ct and displayed ± standard deviation. n = 3. **(b)** Peak assignment to gene-based features for OLDNs called from gel extracted MNase-seq samples. Peaks were called using nucleR from replicate-merged bam files that were bioinformatically filtered to fragment sizes between 230–270 bp (n = 3 per condition, peak width = 250 bp) [41]. **(c)** Putative OLDNs visualized at RNAPII binding sites, sorted by RNAPII occupancy. RNAPII ChIP-seq from GSE98605 [45]. n = 3 merged replicates per condition, shown as a single heatmap. 43,152/45,518 RNAPII ChIP-seq peaks directly overlapped a called “consensus” OLDN peak (94.8%). Gel-extracted libraries were used for these analyses and size-selected for fragments between 230–270 bp. Data were analyzed for significance using a Friedman test (p < 0.001, q < 0.001). **(d)** Metaplots of OLDN occupancy visualized over ChIP-seq peaks for BRG1 (top, GSE64825 [48]) and SNF2H (bottom, GSE123670 [49]). n = 3 replicates per condition. Shaded area indicates standard error. Samples were gel-extracted between 200–300 bp and bioinformatically size-selected for fragments between 230–270 bp. **(e)** Metaplots and heatmaps of OLDN occupancy at TSSs for genes with altered expression in *Smarca4* conditional KO (GSE98469 [45]). Genes are clustered by log_2_ fold change of RNAseq expression as follows: up following knockout (≥ 0.25), unchanged (-0.25 ≤ log_2_ fold change ≤ 0.25), or down following knockout (≤ -0.25). Data were analyzed for significance using Friedman tests for each cluster (p < 0.001 in all cases). All individual comparisons were significant (p < 0.001, q < 0.001) except C2 (*EGFP* KD vs. *Smarca4* KD, low MNase (p = 0.1505, q = 0.0502)) and (*Smarca4* KD vs. *Smarca5* KD high MNase (p = 0.1443, q = 0.0482)). **(f)** Metaplots and heatmaps of OLDN occupancy at TSSs for genes with altered expression in *Smarca4* conditional KO (e; GSE98469 [45]), or *Snf2h* KO (f, GSE112136 [47]). Genes are clustered by log_2_ fold change of RNA-seq expression as in Fig. 3e. Data were analyzed for significance using Friedman tests for each cluster (p < 0.001 in all cases). All individual comparisons were significant (p < 0.001, q < 0.001) except *EGFP KD* vs. *Smarca4* KD under high MNase digestion (p = 0.5388 (C1), 0. 8128 (C2), 0.0454 (C3), q = 0.1798 (C1), 0.2712 (C2), 0.0152 (C3).

Given the strong in vitro evidence for OLDN formation through nucleosome remodeler action [18–21], we attempted to determine whether individual disruption of two nucleosome remodeling complexes, esBAF and ISWI, would alter OLDN occupancy in ES cells. To address this, we performed individual esiRNA-mediated knockdown of the ATPase subunits, *Smarca4* (encoding BRG1) or *Smarca5* (encoding SNF2H), using *EGFP* as an unexpressed control esiRNA target as previously described [68], followed by MNase-seq. Parallel MNase-seq libraries were generated as detailed above (Fig. 1d). We verified effective knockdown for three independent replicates through RT-qPCR using RNA extracted from the same pool of cells used for MNase-seq experiments (Fig. 3a). We identified a reduction of > 70% for the transcripts encoding BRG1 (*Smarca4*) and SNF2H (*Smarca5*), demonstrating effective RNA depletion of each ATPase. We validated the reproducibility of our MNase-seq experiments through a principal component analysis of genome-wide 10 kb bins (Fig. S3a). As expected, the gel-extracted and non-gel-extracted libraries separated into two groups but did not separate based on the individual knockdowns. To address the reproducibility of the experiments more directly, we plotted individual replicates over TSSs (Fig. S3b,d) and gene-distal DHSs (Fig. S3c,e) and observe similar profiles for individual replicates.

To determine whether any global changes in OLDN localization were observed upon remodeler depletion, we called OLDN peaks using nucleR [41] and used HOMER [42] to assign these peaks to the following gene-based classifications: promoters (defined as 1 kb upstream of annotated TSSs), exons, introns, or intergenic regions; we then plotted the log_2_ enrichment of these features relative to their total genomic prevalence (Fig. 3b). We find that remodeler depletion does not broadly alter the genomic distribution of OLDNs, but that OLDNs are consistently enriched at gene-proximal regions. This genic enrichment of OLDNs is in agreement with our analysis of transcription-sorted OLDN occupancy (Fig. 2d). Because nucleosome remodeling factors tend to be more active at highly expressed genes [27, 43, 44], we hypothesized that OLDN formation may occur through combined action of transcription by RNA Polymerase II (RNAPII) and nucleosome remodeling factors. We therefore visualized the extracted MNase-seq data over previously published RNAPII ChIP-seq peaks (Fig. 3c, data from GSE98605) [45]. We found that RNAPII binding is highly correlated with OLDN occupancy, to a greater degree than either nucleosome remodeling factor we examined (Fig. 3d-f), further supporting a transcription-dependent manner of OLDNs formation.

To assess the contribution of nucleosome remodeling factors to OLDN formation more directly, we visualized the MNase-seq data over TSSs, sorted by published RNA-seq comparing *Smarca4* knockout and wildtype ES cells (data from GSE98605) [45]. We ranked expressed genes by log_2_ fold change assigned by DESeq2 [46] and plotted OLDNs over genes in this order, clustered by log_2_ fold change (C1 > 0.25, C2 between 0.25 and -0.25, and C3 < -0.25; Fig. 3e). Intriguingly, the genes that are most sensitive to *Smarca4* knockout (those in clusters 1 and 3) also displayed the highest OLDN occupancy across all experimental conditions. We performed similar analyses on published RNA-seq data comparing *Smarca5* knockout and wildtype ES cells (data from GSE112136) [47] (Fig. 3f); however, we did not identify the same correlation between log_2_ fold change and OLDN occupancy, suggesting that OLDN occupancy may be less reliant on SNF2H-regulated gene expression than BRG1-regulated gene expression. To further interrogate the relationship between OLDN formation and these nucleosome remodelers, we analyzed public ChIP-seq data for BRG1 and SNF2H binding in ES cells (GSE64825 [49], GSE123670 [45], Fig. 3d). At BRG1 peaks, *Smarca4* knockdown led to a slight increase in OLDN occupancy, suggesting a role for esBAF in clearing OLDNs, rather than creating them (Fig. 3d, top). At SNF2H peaks, we obtained conflicting results, where we observe consistent increases in OLDN occupancy under low MNase conditions but decreased OLDN occupancy under high MNase conditions (Fig. 3d, bottom). We suspect this trend is due to a combination of indirect effects of *Smarca5* knockdown, overexpression-dependent SNF2H ChIP-seq, and differing library intensities resulting from these factors; however, these conflicting trends complicate the interpretation of OLDN regulation by SNF2H.

In summary, a strong determinant of genic OLDN occupancy appears to be RNAPII binding (correlated with nascent transcription, Fig. 2d). While OLDNs are formed at BRG1-bound regions (Fig. 3d), OLDN presence is slightly increased at BRG1-bound sites after *Smarca4* knockdown, suggesting this trend may be the result of nucleosome remodeler action at highly transcribed genes or impaired clearance of OLDNs by esBAF.

### esBAF and ISWI regulate fragile nucleosome occupancy

**Fig. 4:**
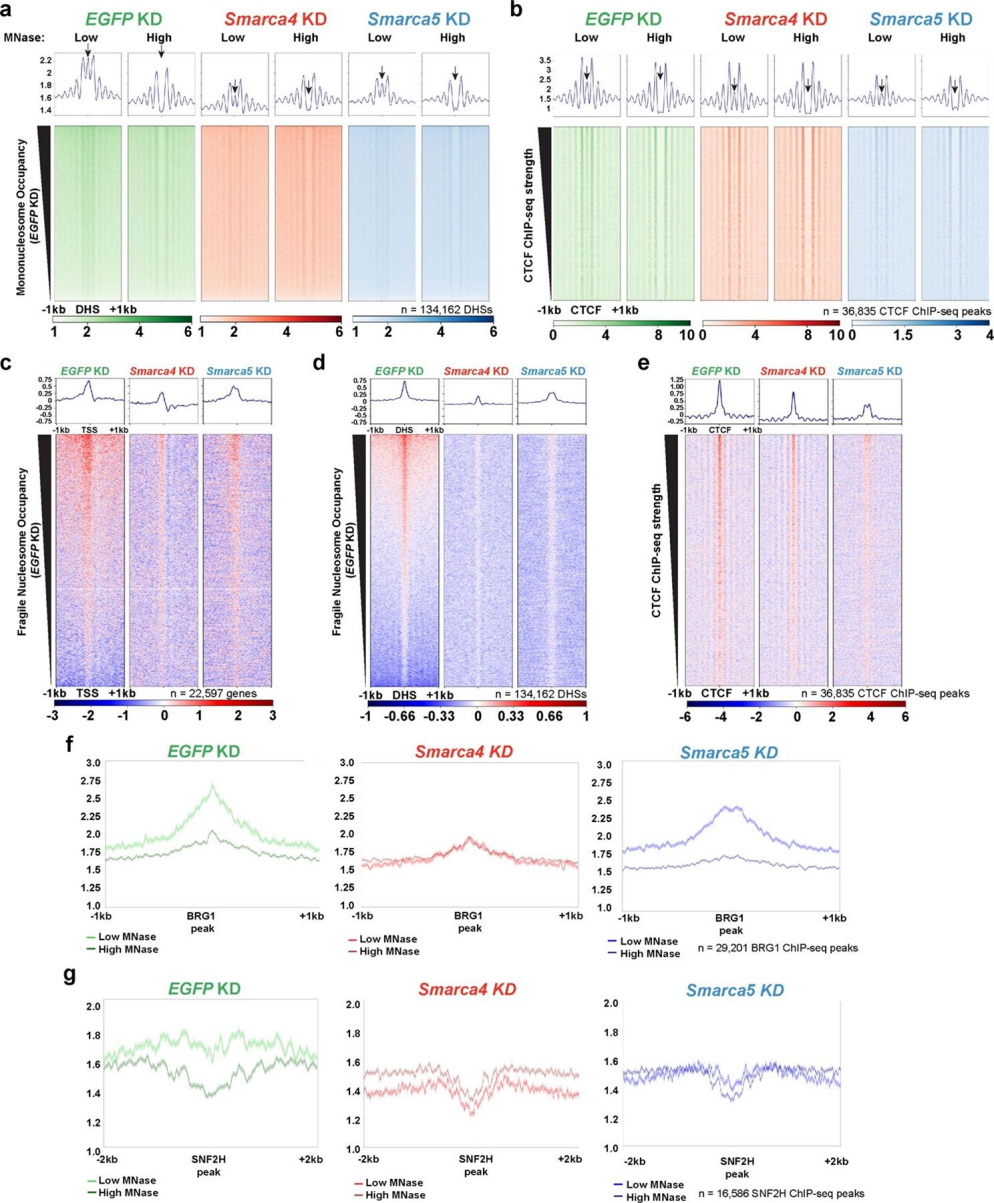
**Fragile nucleosomes are present at promoters, gene-distal DHSs, and CTCF binding sites in mammalian cells and are altered in both BRG1 and SNF2H depletion. (a)** Mononucleosome-sized reads (bioinformatically size-selected for fragments between 135–165 bp) from MNase-seq experiments (non-gel extracted) in *EGFP* knockdown (left), *Smarca4* KD (middle), or *Smarca5* KD (right) over gene-distal DNaseI hypersensitive sites (DHSs, GSM1014154 [37, 58]). n = 3 merged replicates per condition. Data were analyzed for significance using a Friedman test (p < 0.001, q < 0.001). **(b)** As in a, but visualized over CTCF binding sites (from GSE11431 [59]). Data were analyzed for significance using a Friedman test (p < 0.001, q < 0.001). **c-e.** Fragile nucleosome occupancy at TSSs (c), gene-distal DHSs (d), and CTCF binding sites (e), ±1 kb. Fragile nucleosome occupancy was calculated by subtracting occupancy of merged high MNase replicates from occupancy of merged low MNase replicates (n = 3, non-gel-extracted libraries, bioinformatically selected for fragments between 135–165 bp). Data were analyzed for significance using two-tailed Wilcoxon matched-pairs tests comparing the differences between samples with high and low MNase digestion for each knockdown (p < 0.001). **f-g.** Metaplots depicting mononucleosome occupancy for low (light) and high (dark) MNase conditions over BRG1 binding sites (f, GSE64825 [45]) and SNF2H binding sites (g. GSE1236780 [49]) in *EGFP* knockdown (left), *Smarca4* KD (middle), and *Smarca5* KD (right). Data were processed as in panel a. Data were analyzed for significance using two-tailed Wilcoxon matched-pairs tests comparing the differences between samples with high and low MNase digestion for each knockdown (p < 0.001).

Using the low-digestion (5U) and high-digestion (30U) MNase-seq datasets we generated, we examined another understudied alternative nucleosome structure: the fragile nucleosome, known to occupy promoter regions in *S. cerevisiae*, and characterized to a lesser degree in some metazoans [7, 14–17, 50–54]. These structures are only found under mild MNase treatment, making them nuclease sensitive and hence “fragile” nucleosomes. As the BAF complex homolog RSC has been previously shown to regulate fragile nucleosome and subnucleosome occupancy in *S. cerevisiae* [7, 54], we hypothesized that esBAF may regulate fragile nucleosome occupancy in murine ES cells. We examined the MNase-seq datasets following *Smarca4* or *Smarca5* depletion datasets over gene-distal DHSs (Fig. 4a) and CTCF binding sites (Fig. 4b). We find enrichment of fragile nucleosomes over both gene-distal DHSs (Fig. 4a) and CTCF binding sites (Fig. 4b) in low, but not high, MNase digestion conditions. We observe a marked reduction in fragile nucleosome occupancy at the center of the DHS in *Smarca4* knockdown compared with occupancy in the *EGFP* knockdown samples in low MNase conditions, particularly when compared to flanking nucleosome occupancy (Fig. 4a,b; red). We also observe a reduction in mononucleosome occupancy at fragile nucleosome-bound loci following *Smarca5* knockdown, although occupancy flanking gene-distal DHSs is also strongly reduced, complicating the interpretation (Fig. 4a,b; blue). To confirm that these MNase-sensitive particles do indeed contain histones, we performed MNase-ChIP-seq and MNase-ChIP-qPCR targeting histone H3 in WT cells or *EGFP* (control) knockdown (Fig. S4). These experiments demonstrate enrichment of histone H3 above IgG controls at promoter regions, gene-distal DHSs, and CTCF ChIP-seq peaks using low MNase conditions in WT and *EGFP* KD samples, where H3 occupancy is reduced in high MNase digestion conditions (Fig. S4a,b). Further, MNase-ChIP-qPCR results demonstrate that H3 occupancy at each location is partially dependent on ATPase expression (Fig. S4d-f).

Our data are consistent with previous fragile nucleosome profiling experiments, in which only low MNase amounts result in nucleosome profiles at certain loci, including promoter regions and CTCF binding sites [14–17]. To view these MNase-sensitive fragile nucleosomes more directly, we subtracted mononucleosome occupancy after high MNase digestion from occupancy after low MNase digestion, resulting in MNase-sensitive nucleosome-sized particles. We visualized these fragments over promoters (Fig. 4c), gene-distal DHSs (Fig. 4d), and CTCF ChIP-seq peaks (Fig. 4e). At each of these loci, we identified MNase-sensitive fragments, with reduced occupancy following depletion of either *Smarca4* or *Smarca5*, suggesting that nucleosome remodeling factors may create fragile nucleosomes.

Having identified a reduction in fragile nucleosome occupancy upon *Smarca4* depletion at established (promoter, CTCF-bound) and novel (gene-distal DHS) fragile nucleosome-bound genomic locations, we next examined whether this change occurs over other genomic locations, including nucleosome remodeling factor and pluripotency factor binding sites. Using published ChIP-seq datasets [49, 50], we first visualized these MNase-seq data over BRG1 peaks, identifying a much stronger enrichment of mononucleosome occupancy under low-MNase conditions than high-MNase conditions, which we interpret to be the presence of fragile nucleosomes (Fig. 4f). After *Smarca4* knockdown, however, nucleosome occupancy over BRG1 binding sites is virtually indistinguishable between the MNase conditions (Fig. 4f), suggesting that these fragile nucleosomes are dependent on the remodeling action of BRG1. As a control, we also visualized *Smarca5* knockdown MNase-seq data over BRG1 ChIP-seq peaks (Fig. 4f), identifying a similar profile as observed in the control MNase-seq data (Fig. 4f); we therefore do not interpret the loss of fragile nucleosome occupancy in Fig. 4a-e to be due to a global reduction in nucleosome occupancy due to the effects of remodeler knockdown. We similarly examined our MNase-seq data over SNF2H ChIP-seq peaks [49] and identified nucleosome enrichment in low MNase conditions above high MNase conditions for the *EGFP* (control) knockdown MNase-seq, again suggesting the low MNase profile reflects fragile nucleosomes (Fig. 4g). However, in both *Smarca4* and *Smarca5* knockdown samples, low MNase and high MNase digestion conditions are very similar, suggesting a loss in fragile nucleosome occupancy over these SNF2H binding sites when either BRG1 or SNF2H is depleted. Because SNF2H binding was profiled using *Smarca5* overexpression, the unexpected effect of *Smarca4* knockdown may be due to irregular SNF2H peaks or due to co-binding by BAF and ISWI (e.g., at gene-distal DHSs).

Because of the marked effect of *Smarca4* knockdown on fragile nucleosomes over gene-distal DHSs, and because of the established requirement for BRG1 in ES cell pluripotency [56–58], we next examined whether fragile nucleosomes are observed at sites bound by the master pluripotency factors OCT4, SOX2, KLF4, and NANOG. As the majority of these factors’ binding sites are gene-distal, we hypothesized that fragile nucleosomes would be observed at these sites, and that *Smarca4* and *Smarca5* depletion would lead to a reduction of fragile nucleosome occupancy. Indeed, when visualized over OCT4 (Fig. S5a), SOX2 (Fig. S5b), KLF4 (Fig. S5c), and NANOG (Fig. S5d) binding sites a decrease in nucleosome occupancy directly over the binding site under low, but not high, MNase digestion conditions. Interestingly, occupancy of flanking nucleosomes also decreased under both MNase conditions following either *Smarca4* or *Smarca5* depletion, underscoring the effect of remodeler knockdown on well-positioned nucleosomes as well as fragile nucleosomes.

To control for possible confounding variables, we examined our MNase-seq data over CHD1 binding sites (ChIP-seq from GSE64825 [48]), which we hypothesized would not be affected by remodeler knockdowns, due to the established role of CHD1 in regulation of nucleosome occupancy co-transcriptionally through gene bodies [61–64]. Indeed, we saw little to no enrichment of low MNase specific mononucleosome occupancy, inferred as fragile nucleosomes, over CHD1 binding sites (Fig. S5e-g). We also saw no change in mononucleosome occupancy upon either remodeler depletion over CHD1 binding sites, providing further evidence that the changes observed over BRG1-bound locations (Fig. 4f) are specific to BRG1-mediated fragile nucleosomes, rather than global depletion effects.

### esBAF oppositely regulates fragile nucleosomes and overlapping dinucleosomes

**Fig. 5:**
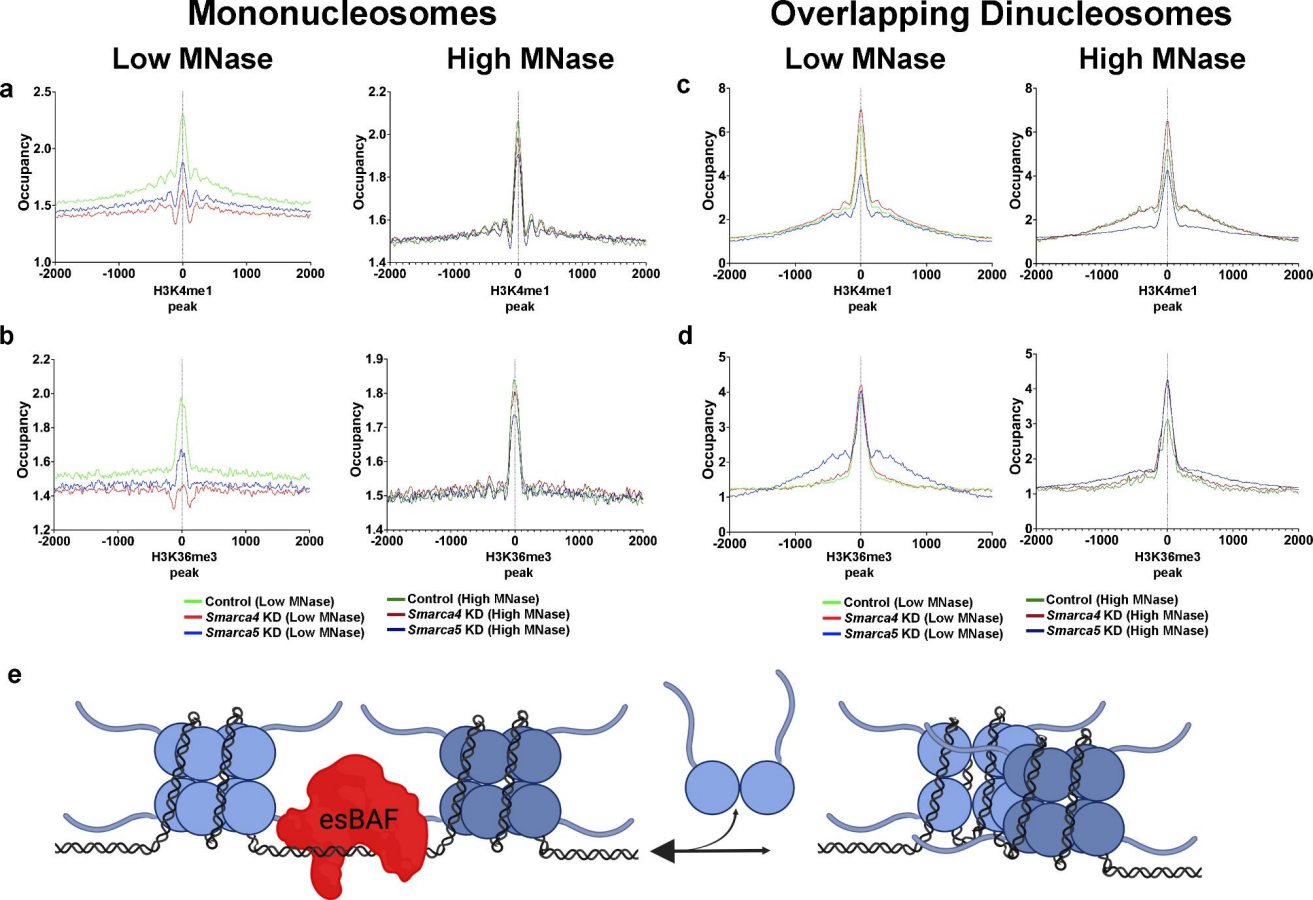
**The esBAF complex oppositely regulates occupancy of fragile nucleosomes and overlapping dinucleosomes. a-b.** Mononucleosome occupancy visualized over H3K4me1 (a, GSE31039 [37, 58]) and H3K36me3 ChIP-seq peaks (b, GSE31039 [37, 58]). n = 3 merged replicates per condition. Samples were not gel-extracted but were bioinformatically selected for fragments between 135–165 bp. **c-d.** Overlapping dinucleosome occupancy following *Smarca4, Smarca5*, or *EGFP* control knockdown as in a-b. n = 3 merged replicates per condition. Samples were gel-extracted between 200–300 bp and bioinformatically selected for fragments between 230–270 bp. **e.** Proposed model integrating regulation of OLDNs and fragile nucleosomes by esBAF.

We observed consistent effects of *Smarca4* knockdown on fragile nucleosomes and on OLDNs at BRG1 binding sites and therefore wanted to understand if there may be a relationship between the two nucleosome structures mediated by esBAF. While we observed a modest correlation between the transcriptional effect of *Smarca4* knockout (Fig. 3f) and OLDN occupancy, we did not identify consistent effects of remodeler depletion on TSS-proximal OLDNs. Therefore, we examined our MNase-seq data at locations containing two histone posttranslational modifications not traditionally associated with promoters: H3K4me1, which canonically marks enhancers, and H3K36me3, which is placed on elongating genes co-transcriptionally. Due to the enrichment of fragile nucleosomes at gene-distal DHSs (Fig. 4a,d), many of which are enhancers, we anticipated a reduction in fragile nucleosome occupancy at H3K4me1 sites upon remodeler depletion. As most OLDN peaks we identified were genic (Fig. 3b), we expected to see effects of *Smarca4* depletion on OLDNs at H3K36me3 sites. We visualized the MNase-seq data over H3K4me1 (Fig. 5a, c) and H3K36me3 (Fig. 5b, d) ChIP-seq peaks (both from GSE31039 [37, 58]), plotting both the mononucleosome (Fig. 5a, b) and OLDN (Fig. 5c, d) size classes at these loci. At both histone modification ChIP-seq peaks, we see a consistent trend upon *Smarca4* knockdown: mononucleosome occupancy is decreased when compared with *EGFP* (control) knockdown MNase-seq data, while OLDN occupancy is increased. The effect of *Smarca4* knockdown on mononucleosomes is stronger under low MNase conditions, while the opposite is true of OLDN occupancy; effects are more muted under low MNase conditions than high MNase conditions (Fig. 5a-d). Intriguingly, mononucleosomes in *Smarca5* knockdown mirror those in *Smarca4* knockdown, but OLDN occupancy is increased at H3K36me3 peaks and decreased at H3K4me1 peaks when compared with *EGFP* knockdown. We interpret this trend as signifying a primary role for nucleosome remodelers in clearing OLDNs created by genic transcription, though remodeling action at enhancer loci may have a secondary role in creating OLDNs. Based on these opposite trends for fragile nucleosomes and overlapping dinucleosomes, we propose a model in which esBAF facilitates both the formation and clearance of OLDNs from fragile nucleosomes (Fig. 5e).

## Discussion

In this study, we mapped both OLDN and fragile nucleosome enriched locations in murine embryonic stem cells. We find both alternative nucleosomes are enriched at TSSs, gene-distal DHSs, and CTCF binding sites. Furthermore, we explored the role of two nucleosome remodeling factors, BRG1 and SNF2H, catalytic subunits for esBAF and ISWI, respectively. Whereas esBAF is responsible for creating or maintaining fragile nucleosome occupancy, the complex may prevent OLDN occupancy at both genic (H3K36me3-marked) and non-genic (H3K4me1-marked) loci (Fig. 5). These opposing trends on alternative nucleosome structures may be related and we propose that fragile nucleosomes and OLDNs may result from the clearance of one another by nucleosome remodelers (Fig. 5e). In line with prior reports, including MNase-seq, MNase-SSP, and chemical cleavage mapping studies, we recapitulated MNase-sensitive fragile nucleosome occupancy near TSSs and CTCF binding sites, along with novel identification at gene-distal DHSs [7, 14, 15, 66]. Given the extensive overlap between regions that harbor fragile nucleosomes and overlapping dinucleosomes (including DHSs, promoters, CTCF binding sites, and H3K4me1 sites), these alternative nucleosome structures are likely similarly regulated, if not directly co-regulated. As fragile nucleosomes are characterized by a smaller, subnucleosome-sized DNA footprint [6, 7], they may be created through the splitting of a 14-mer OLDN into a canonical octasome and a fragile subnucleosome. We find that both fragile nucleosomes and OLDNs are affected by *Smarca4* knockdown. While both fragile nucleosomes and OLDNs may be independently regulated by the esBAF complex; we propose that these structures’ regulation may be interrelated due to the consistently opposing results on occupancy observed upon *Smarca4* knockdown. Future studies of alternative nucleosome dynamics at single loci harboring both OLDNs and fragile nucleosomes will illuminate whether their opposing regulation by esBAF is mechanistic or correlative in nature; if the former, these experiments may shed light on an unstudied mechanism of gene regulation by esBAF.

## Conclusions

In this study, we find that OLDNs are enriched at gene-regulatory regions in murine ES cells, including promoters and gene-distal DHSs (Fig. 1), qualitatively correlating with both mononucleosome levels and transcription (Fig. 2). OLDN occupancy at TSSs is correlated with altered gene expression after *Smarca4* knockout, and depletion of BRG1 leads to increased OLDN occupancy, suggesting a repressive role for esBAF in regulating OLDNs (Fig. 3). In contrast, we find that *Smarca4* knockdown abrogates fragile nucleosome occupancy at esBAF binding sites and at regions marked by DNaseI hypersensitivity or CTCF binding (Fig. 4). We find that these opposing trends hold true at both genic (H3K36me3-marked) and gene-distal (H3K4me1-marked) regions, suggesting a universal mechanism of OLDN regulation by esBAF (Fig. 5). Therefore, in this study we have identified opposing roles for the esBAF catalytic subunit BRG1 in regulation of fragile nucleosomes and overlapping dinucleosomes.

Our in vivo data validate prior in vitro experiments suggesting a role for the BAF complex in OLDN regulation [18–20], but our data do not conclusively identify how OLDNs are created in vivo. Moreover, the question remains whether OLDNs and fragile nucleosomes participate in regulatory processes—perhaps through occlusion of factor binding (e.g., due to the missing nucleosome acidic patch)—or if they are simply transient byproducts of nucleosome mobilization. Our data show that esBAF preferentially clears OLDNs, suggesting that the latter may be the case; however, genomic characterizations at BRG1*-*dependent OLDN loci are necessary to truly determine whether OLDNs function in genome regulation. Additionally, genomic experiments testing inhibition of RNAPII may illuminate if there is a polymerase-based mechanism for OLDN formation, or simply correlation with transcription due to remodeler action. We hypothesize that there is a RNAPII-based mechanism, as TSS-proximal OLDNs are more prominent under high MNase digestion, while gene-distal OLDNs are more prominent under low MNase digestion due to lower transcriptional rates than genic regions, and therefore less stable OLDN occupancy.

Fragile nucleosomes, on the other hand, are absent from esBAF binding sites after *Smarca4* knockdown (Fig. 4f), suggesting that esBAF creates fragile nucleosomes through the remodeling action of BRG1. While it remains to be seen whether fragile nucleosomes directly contribute to genome regulation, their partly esBAF-dependent presence at pluripotency factor binding sites (Fig. S5), many of which are esBAF-regulated gene-distal regulatory elements, suggest that they may participate in distal gene regulation.

## Materials and methods

### Materials availability

No plasmids or cell lines were generated in this study, but materials are available on request. All resources generated in this study must be acquired via a Material Transfer Agreement granted by the University of Pittsburgh.

### Cell lines

ES-E14TG2a (E14) embryonic stem cells from male *Mus musculus* origin (RRID:CVCL9108; [67]; a gift from the Fazzio lab) were cultured in medium at 37 °C/5% CO_2_ on 0.2% gelatin pre-coated plates (feeder free). Cells were cultured in DMEM base medium (Gibco) with 10% fetal bovine serum (Sigma, 18N103), 1X nonessential amino acids (Gibco), 2mM L-glutamine (Gibco), β-mercaptoethanol (Acros Organics), and 1000U/mL leukemia inhibitory factor (LIF). Cells were passaged every 48 h using trypsin (Gibco) and split at a ratio of ~ 1:8 with fresh medium. Routine anti-mycoplasma cleaning was conducted (LookOut DNA Erase spray, Sigma) and cell lines were screened by PCR to confirm no mycoplasma presence.

### RNAi knockdowns

Endoribonuclease-digested short interfering RNAs (esiRNAs) were generated as previously described [68–70]. Briefly, DNA sequences were identified within the target genes and screened for unique nucleotide sequence via DEQOR [71]. siRNAs were in vitro transcribed using T7 polymerase using cDNA collected from wildtype murine ES cells as a template. siRNAs were then digested with ShortCut RNase III (NEB) into esiRNAs, which were then purified using a PureLink RNA Mini Kit (Invitrogen). Transient transfections were performed on 10 cm plates using 25 µL of Lipofectamine 3000 (ThermoFisher) and 3.5 µg of esiRNAs for 48 h and validated by RT-qPCR.

### RNA isolation

TRIzol reagent (Invitrogen) was used to isolate total RNA according to manufacturer’s protocol. Quality and quantity were assessed via NanoDrop (ThermoFisher) and RNA was snap-frozen in liquid nitrogen and stored at -80 °C.

### Reverse transcription and quantitative PCR (RT-qPCR)

cDNA was synthesized from 1 µg of RNA with random hexamers (Promega) with homemade reverse transcriptase. cDNA was used as a template in qPCR reactions using 2X SYBR FAST mix (KAPA) on a Lightcycler 96 (Roche) with 5 µM specific primers (see Supplementary Table 1). Slight differences in RNA concentration were controlled for using ∆∆Ct normalization to *Gapdh* transcript abundance. Technical replicates represent the average of three individual qPCR reactions for each target/condition group. Error bars shown represent the standard deviation of qPCR replicates. Biological replicates (plotted separately in Fig. 3a) represent independently transfected and harvested cells, while technical replicates represent individual qPCR reactions from the same cDNA pool.

### Micrococcal nuclease digestion coupled with deep sequencing

MNase-seq was performed as previously described [28]. esiRNA-transfected E14 ES cells (10^7^) were pelleted and rinsed in Dulbecco’s phosphate-buffered saline (Gibco). Cells were then crosslinked with 1% formaldehyde in PBS for 10 min at room temperature in a volume of 8 mL, inverting every 3 min. After adding 2 mL 2.5 M glycine, cells were mixed thoroughly by repeated inversion and left for 5 min at room temperature to quench the reaction. Cells were then pelleted and rinsed twice with PBS, snap-frozen in liquid nitrogen, and stored at -80 °C. Cells were thawed, re-suspended in cell lysis buffer (10 mM Tris pH 7.5, 10 mM NaCl, 2 mM MgCl_2_, 0.5% NP-40, freshly-added EDTA-free protease inhibitors (Pierce) and 3 µM CaCl_2_) at a proportion of 3 mL buffer for 10 M cells, and left to rotate for 15 min at 4 °C. Micrococcal nuclease (MNase) enzyme (Takara) was aliquoted into three pre-chilled tubes in amounts of 0U, 10U (low MNase), or 60U (high MNase). Lysed cells were equally added to each prepared tube and chromatin was digested for 5 min at 37 °C. The MNase digestion reaction was chelated with 10 mM EDTA at room temperature, and samples were treated with RNase A for 2 h at 37 °C before adding 0.01% SDS and Proteinase K and incubating at 55 °C overnight to reverse crosslinks. Samples were purified using a phenol-chloroform-isoamyl alcohol (PCI) extraction, followed by chloroform extraction and ethanol precipitation, then resuspended in nuclease-free water. One µg of purified chromatin was set aside for library preparation and deep sequencing (for traditional, unextracted MNase-seq analysis), and the remainder of each sample was run on ethidium bromide-stained 1.5% agarose gel to verify sample quality and isolate a parallel overlapping dinucleosome enriched MNase-seq sample. The section of the gel corresponding to ~ 200–300 bp fragments was gel-extracted (Qiagen) to enrich for putative overlapping dinucleosomes. Paired-end libraries of MNase-digested DNA were prepared as described previously [28]. Briefly, phosphate ends were removed with CIP (NEB), and samples were end-repaired, A-tailed, and adaptor-ligated for Illumina sequencing as described [28]. Between each step, DNA was purified with PCI extraction and ethanol precipitation. After adaptor ligation, DNA was purified with Agencourt AMPure XP beads (Beckman Coulter) and PCR amplified with HiFi polymerase (KAPA). Libraries were purified with a DNA Clean & Concentrator kit (Zymo) and quantified using a Qubit (Invitrogen). Libraries were sequenced on a NextSeq2000 at the HSSC Sequencing Core facility, located at the Children’s Hospital of Pittsburgh.

### MNase-seq data analysis

Paired-end fastq files were trimmed to 25 bp and mapped to the mm10 genome with bowtie2 (using the options -q -N 1 -X 1000 –no-unal) [72]. Mapped reads were duplicate-filtered using Picard [73] and filtered for mapping quality (MAPQ ≥ 10) and unpaired reads using SAMtools [74]. Reads were then sorted into OLDN- (230–270 bp), nucleosome- (135–165 bp), and subnucleosome-sized fragments (100–130 bp) using a custom awk script and SAMtools [74]. Sized reads were converted to bigWig files using deepTools bamCoverage (options -bs 1 --smoothLength 10 --normalizeUsing RPGC, --effectiveGenomeSize 2,308,125,349 -e --centerReads) [75]. Peaks representing OLDNs were called using nucleR (option --width 250) from reads that were merged between replicates using SAMtools[61, 93]. “Consensus” peaks, defined as being represented in three or more out of 6 replicate-merged bam files, were called using HOMER mergePeaks (options -d given) [42]. Heatmaps were generated using deepTools computeMatrix (options -bs 10 --missingDataAsZero) and plotHeatmap and plotProfile [75]. Metaplots were plotted in PRISM 9 using the matrices generated by plotProfile. Gene-distal DHSs were identified by subtraction of annotated mm10 or hg38 transcription start sites from master lists of DNaseI hypersensitive sites (ENCODE consortium (GSM1014154, GSE90432) [37, 58].

To determine if nucleosome remodeling factor knockdowns had a significant effect on OLDN occupancy, we applied Friedman tests to generated heatmap data matrices, correcting for multiple comparisons using the Benjamini, Krieger, and Yekutieli method with an FDR/q-value cutoff of 0.001. For MNase-sensitive particle occupancy, we applied two-tailed Wilcoxon matched-pairs signed rank tests to the differences between occupancy of individual regions under low and high MNase digestion, comparing each knockdown back to the EGFP KD control, with a p-value cutoff of 0.001. All statistical calculations were performed in GraphPad PRISM 9.

### MNase-ChIP

MNase-ChIP was performed as described [76]. Briefly, cells were crosslinked using formaldehyde, lysed and incubated with MNase for 5 min. The reaction was quenched using EDTA and samples were pre-cleared with protein A beads (NEB) in complete immunoprecipitation buffer (20mM Tris-HCl pH[7.5], 1 M NaCl, 2 M EDTA, 0.1% Triton X-100 with fresh protease inhibitors added). Input samples were taken from cleared chromatin. Pre-coupled antibody-protein A beads were added to cleared chromatin and incubated overnight at 4 °C with rotation. Antibodies used were IgG (Abcam: ab37415, lot: GR3208186-1) and H3 (Abcam: ab1791, lot: GR3428015-1). Bead-bound samples were washed twice with low salt wash (20mM Tris HCl pH [8.0], 2mM EDTA, 150mM NaCl, 1% Triton X-100, 0.1% SDS) and then twice with high salt wash (20mM Tris HCl pH [8.0], 2mM EDTA, 500mM NaCl, 1% Triton X-100, 0.1% SDS). Samples were eluted at 65 °C for 1.5 h in elution buffer (100mM NaHCO_3_, 1% SDS) and cleaned using AMPureXP beads (Beckman Coulter). Cleaned DNA was used as a template for qPCR or for input into library builds. Library preparation was performed using NEB Next II Ultra DNA kit with 14 cycles of PCR amplification. Libraries were sequenced on a NextSeq2000 at the HSSC Sequencing Core facility, located at the Children’s Hospital of Pittsburgh.

### MNase-ChIP-qPCR

MNase-ChIP samples were prepared as described above. Eluted and cleaned DNA was used as a template in qPCR reactions using 2X SYBR FAST mix (KAPA) on a Lightcycler 96 (Roche) with 5 µM specific primers (see Supplementary Table 1). Technical replicates presented in Fig. S4 represent the average of three individual qPCR reactions for each target/condition group. Error bars shown represent the standard deviation of qPCR replicates.

### MNase-ChIP-seq data analysis

Paired-end fastq files were trimmed to 25 bp and mapped to the mm10 genome with bowtie2 (using the options -q -N 1 -X 1000 –no-unal) [72]. Mapped reads were filtered for mapping quality (MAPQ ≥ 10) and unpaired reads using SAMtools [74]. Filtered reads were converted to bigWig files and normalized to 1x coverage using deepTools bamCoverage (options -bs 5 --smoothLength 20 --normalizeUsing RPGC, --effectiveGenomeSize 2,308,125,349 -e --centerReads) [75]. Normalized bigwig files were controlled for nonspecific chromatin pulldown by subtracting IgG control occupancy from H3 occupancy and plotted using deepTools computeMatrix (options -bs 10 --missingDataAsZero) and plotHeatmap and plotProfile [75].

### RNA-seq data analysis

Paired-end fastq files were trimmed and filtered using Trim Galore [77], then aligned to the mm10 mouse genome using STAR (options --outSAMtype SAM --outFilterMismatchNoverReadLmax 0.02 --outFilterMultimapNmax 1). Aligned reads were filtered for MAPQ ≥ 7 using SAMtools [74]. Feature counts were generated using subread featureCounts (options -s 0/2 -p -B) for annotated genes based on Gencode vM25 coordinates. Feature counts were imported to R and downstream analysis was conducted using DESeq2 with apeglm log-fold change shrinkage [46, 78]. DESeq2 results were filtered for expression (baseMean ≥ 1).

**Table 1:** Accession numbers and associated manuscripts for published data analyzed for this study

Experiment	Associated Manuscript	Accession Number
MNase-seq		
E14 MNase-seq	Blümli et al. 2021	GSE183278
HeLa MNase-seq	Kato et al. 2017	DRP003456
**ChIP-seq**		
BRG1 ChIP-seq	de Dieuleveult et al. 2016	GSE64825
CHD1 ChIP-seq	de Dieuleveult et al. 2016	GSE64825
SNF2H ChIP-seq	Song et al. 2022	GSE123670
RNAPII ChIP-seq	Hodges et al. 2018	GSE98605
CTCF ChIP-seq	Chen et al. 2008	GSE11431
OCT4 ChIP-seq	Marson et al. 2008	GSE11724
SOX2 ChIP-seq	Marson et al. 2008	GSE11724
KLF4 ChIP-seq	Chen et al. 2008	GSE11431
NANOG ChIP-seq	Marson et al. 2008	GSE11724
H3K4me1 ChIP-seq	Davis et al. 2018	GSE31039
H3K36me3 ChIP-seq	Davis et al. 2018	GSE31039
**RNA-seq**		
SNF2H KO RNAseq	Barisic et al. 2019	GSE112136
BRG1 KO RNAseq	Hodges et al. 2018	GSE98469
**TT-seq**		
E14 TT-seq	Klein et al. 2022	GSE181624
E14 TT-seq	Blümli et al. 2021	GSE183278
**DNase I Hypersensitive Sites**		
E14 DHSs	Davis et al. 2018	GSM1014154
HeLa DHSs	Davis et al. 2018	GSE90432

### Electronic supplementary material

Below is the link to the electronic supplementary material.

Supplementary Material 1

## Data Availability

The datasets develop for this project and supporting the conclusions of this article are available in the NCBI GEO under accession number GSE216057. This paper analyzes existing, publicly available data housed in the NCBI Gene Expression Omnibus (GEO) and the Sequence Read Archive (SRA). The accession numbers for the datasets are listed throughout the manuscript and in Table 1. These accession numbers include: GSE183278, DRP003456, GSE64825, GSE123670, GSE98605, GSE11431, GSE11724, GSE31039, GSE112136, GSE98469, GSE181624, GSM1014154, GSE90432. Any additional information required regarding the data reported in this paper is available from the corresponding author upon request.
