# Correction: The esBAF and ISWI nucleosome remodeling complexes influence occupancy of overlapping dinucleosomes and fragile nucleosomes in murine embryonic stem cells

**DOI:** 10.1186/s12864-023-09806-3

**Published:** 2023-11-21

**Authors:** David C. Klein, Kris Troy, Sarah A. Tripplehorn, Sarah J. Hainer

**Affiliations:** 1 Department of Biological Sciences, University of Pittsburgh, Pittsburgh, PA 15213 USA; 2 Department of Quantitative and Systems Biology, University of California, 95343 Merced, Merced, CA USA

**Correction: BMC Genomics 24, 201 (2023)**

10.1186/s12864-023-09287-4

Following publication of the original article [1], it was reported that there was an error in the caption of Fig. 3.

The caption of Fig. 3 originally read: “**Fig. 3 Overlapping dinucleosome formation is stimulated by the nucleosome remodeler BRG1.**”

The correct Fig. 3 caption is: “**Fig. 3 Overlapping dinucleosome occupancy is dependent on the nucleosome remodeler BRG1**.”

The text describing the individual figure panels a-d was correct and remains unchanged. The original article has been updated.
