# Supplementary material for: The esBAF and ISWI nucleosome remodeling complexes influence occupancy of overlapping dinucleosomes and fragile nucleosomes in murine embryonic stem cells
Source: BMC Genomics. 2023 Apr 13;24:201. doi: 10.1186/s12864-023-09287-4 (PMC10103515; doi:10.1186/s12864-023-09287-4)
Supplement: Supplementary file 1 — Supplementary Material 1 [file 12864_2023_9287_MOESM1_ESM.docx]

**Supplementary Figures**

**
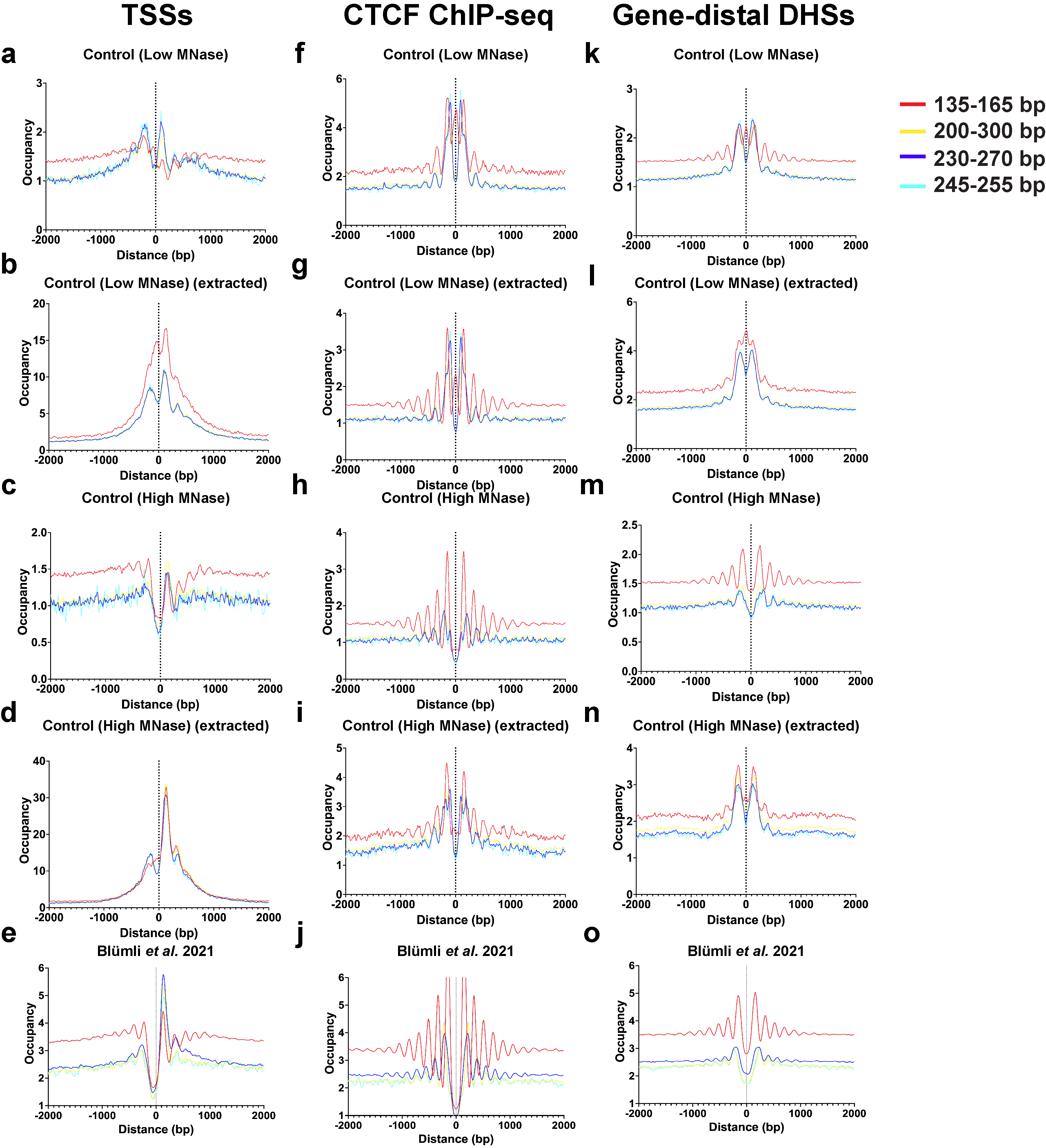
**

**Supplementary Figure 1. Validation of size classes for bioinformatic putative OLDN enrichment.** Reads from our control datasets (a-d, f-I, k-n) and published murine ES cell MNase-seq data from Blümli *et al.* (GSE183278[1], e, j, o), were computationally assigned to mononucleosomes (purple, 135-165 bp), as well as three size classes containing putative OLDN reads (200-300 bp, 230-270 bp, and 245-255 bp). Alternative size classes were plotted at annotated RefSeq Select TSSs (left column), CTCF ChIP-seq peaks (GSE11431[2], middle column), and gene-distal DHSs (GSM1014154[3], right column). n = 2 merged replicates per condition, shown as a single track. Mononucleosome libraries were not gel-extracted, while OLDN libraries were gel-extracted, with the exception of the samples from Blümli *et al* ([1] bioinformatically size-selected between 230-270 bp but not gel-extracted)*.*

**
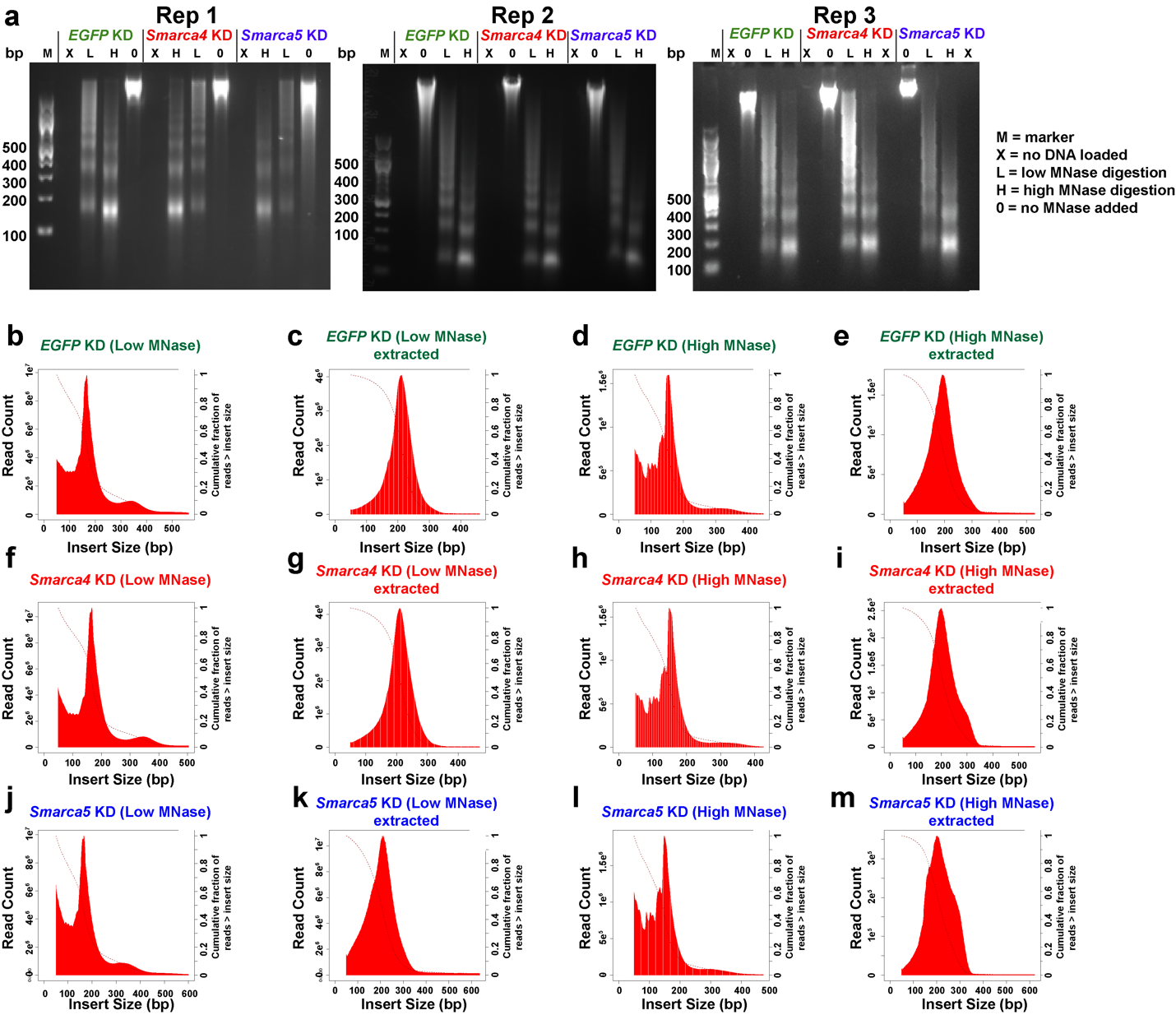
Supplementary Figure 2. MNase-seq quality controls. a.** Ethidium bromide-stained agarose gel images of MNase digested samples. M = 100bp NEB ladder, X = empty well, 0 = no MNase, L = low MNase digestion, H = high MNase digestion. **b-m.** Insert size plots showing read distribution among combined replicates for each knockdown condition. Insert sizes were calculated using Picard[4] after removing duplicate and low-quality reads but before performing any read normalization. Dashed lines indicate fraction of reads greater than insert size (right y-axis). n = 3 merged replicates per condition, shown as a single plot.


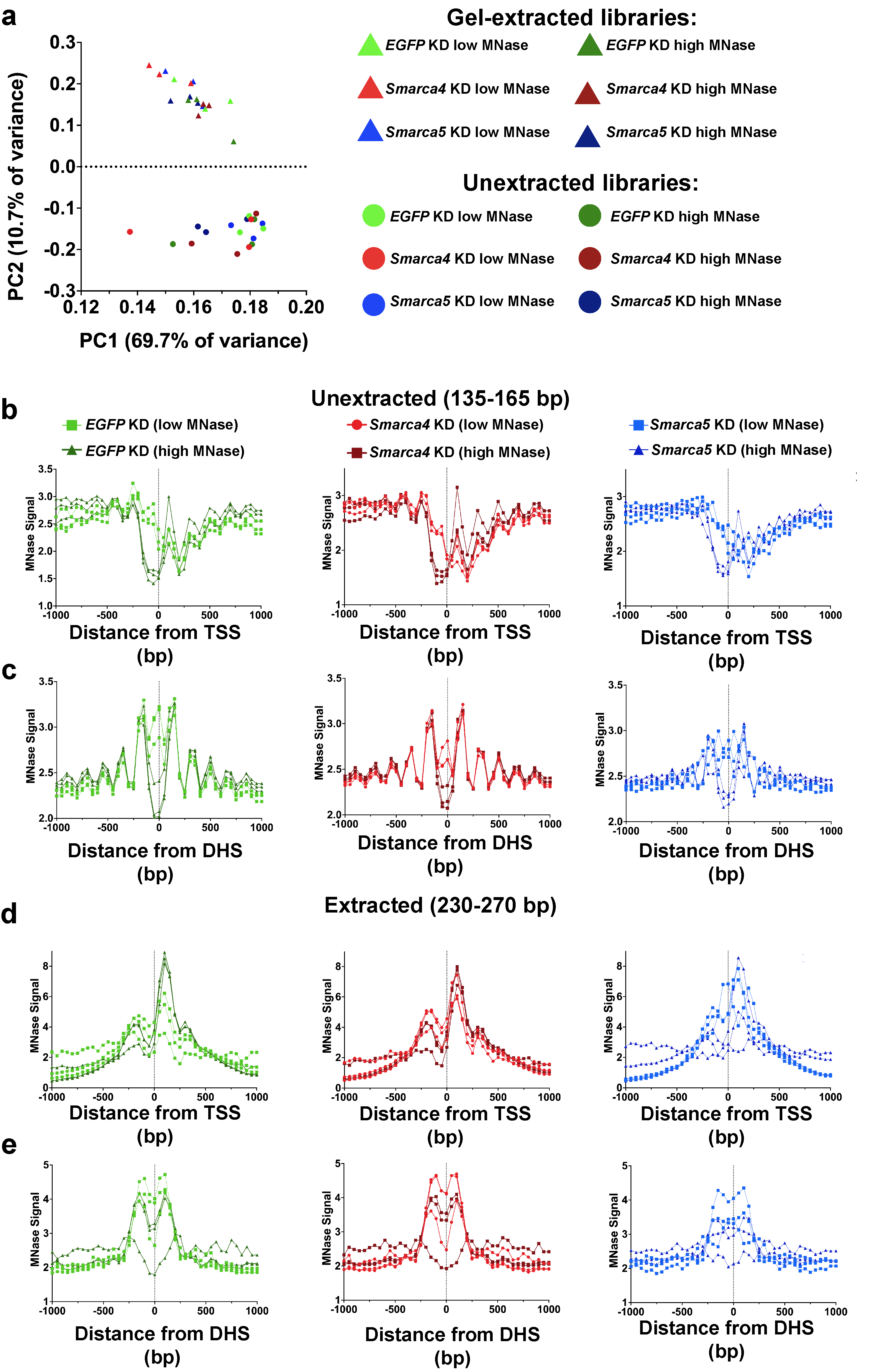
**Supplementary Figure 3. a.** Plotted principal component analysis (PCA) of MNase-seq datasets. **b-e.** Comparison of individual MNase-seq replicate experiments, visualized over annotated mm10 RefSeq Select mRNA TSSs (b, d) and gene-distal DHSs (c, e; from GSM1014154[5]) for unextracted (b-c) and gel-extracted libraries (d-e). Unextracted libraries were bioinformatically size-selected to include mononucleosomal fragments (135-165 bp), while gel-extracted libraries were size-selected to include fragments between 230-270 bp. Each replicate is connected by a single line, and the score for each bin is plotted at 50-bp intervals as a symbol.

**
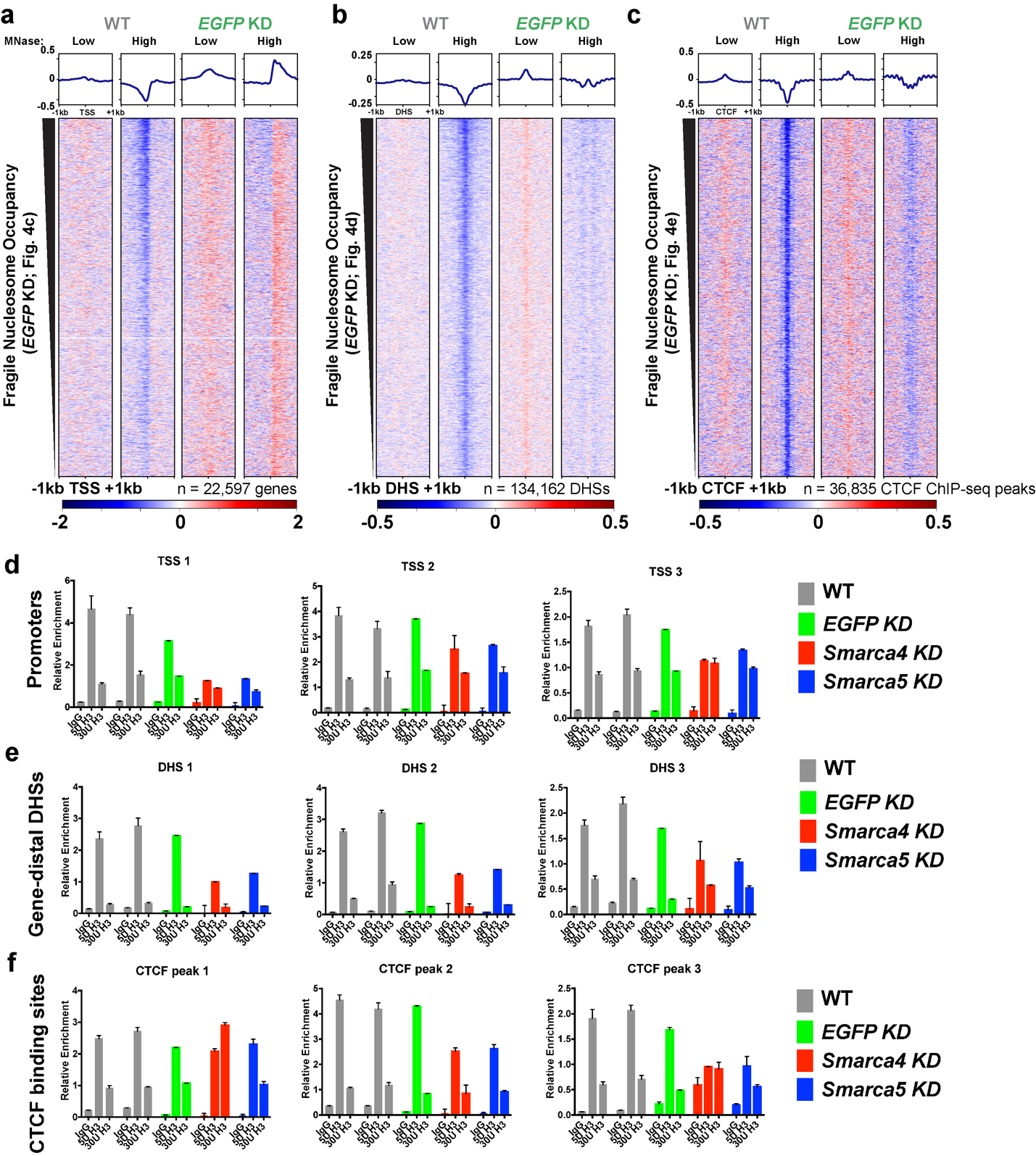
Supplementary Figure 4.** **Regions identified as fragile nucleosomes contain histone H3 in a digestion-dependent manner. a.** MNase-ChIP-seq data plotted over annotated mm10 RefSeq Select TSSs, ± 1kb. Heatmaps represent anti-H3 MNase-ChIP-seq data minus occupancy of IgG control and are sorted by difference in nucleosome occupancy between high- and low-MNase digestion in Fig 4c-e. For wildtype (WT; without knockdown), n = 2 where data represents the merged replicates; for *EGFP* knockdown, n = 1. MNase-ChIP was performed with low MNase digestion (5U) or high MNase digestion (30U). **b.** as in panel a, but visualized over gene-distal DHSs (from GSM1014154[1]). **c.** as in panel a, but visualized over CTCF ChIP-seq peaks from GSE11431[2]. **d-f:** MNase-ChIP-qPCR targeting histone H3 at three promoter regions (d), three gene-distal DHSs (e), and three CTCF binding sites (f). We performed the experiment in WT cells (without knockdown; gray) and following knockdowns of *EGFP* (green; exogenous control), *Smarca4* (red), and *Smarca5* (blue). All Cq values were made relative to input and plotted with the standard deviation of technical qPCR replicates shown (n = 2 for WT, n = 1 per knockdown). MNase-ChIP was performed with low MNase digestion (5U) or high MNase digestion (30U).

**
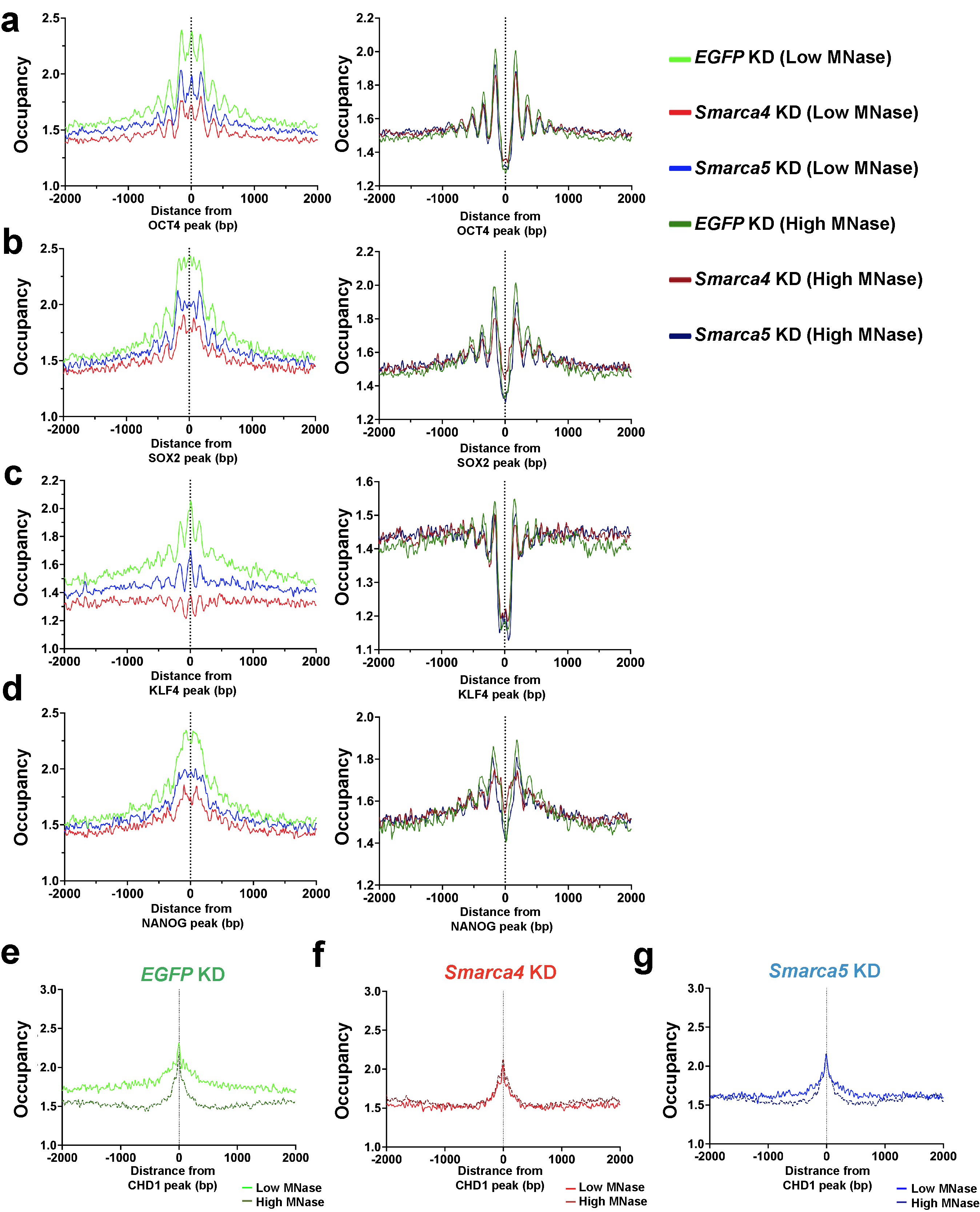
**

**Supplementary Figure 5. Effects of remodeler knockdown on mononucleosome occupancy at pluripotency-associated ChIP-seq peaks. a-d.** Mononucleosome occupancy plotted over ChIP-seq peaks for pluripotency factors OCT4 (a), SOX2 (b), KLF4 (c) and NANOG (d). n = 3 merged replicates per condition, shown as a single track. Experiments performed under low MNase digestion are shown in left plots, while high MNase digestion experiments are shown in right plots. ChIP-seq datasets from GSE11724[8]. **e-g.** Mononucleosome occupancy plotted over CHD1 genomic binding sites. n = 3 merged replicates per condition, shown as a single track. ChIP-seq data from GSE64825[9]. Non-gel-extracted libraries were used for these analyses.

| **esiRNA generation** | **Sequence (5’ - 3’)** |
| --- | --- |
| ***EGFP F*** | GGGCGGGTCGTAAACGGCCACAAGTTCA |
| ***EGFP R*** | GGGCGGGTATGGGGGTGTTCTGCTGGTA |
| ***Smarca4 F*** | GGGCGGGTGACGCCCGACACATTATTGAG |
| ***Smarca4 R*** | GGGCGGGTTGCAGTGGTGGTTTTTCATG |
| ***Smarca5 F*** | GGGCGGGTCAGAATTTGCTTTCAGTTGGAG |
| ***Smarca5 R*** | GGGCGGGTAGCAACAGCCGATTTGTAGTC |
|  |  |
| **qPCR primers:** |  |
| ***Gapdh F*** | TTGATGGCAACAATCTCCAC |
| ***Gapdh R*** | CGTCCCGTAGACAAAATGGT |
| ***Smarca4 F*** | GGACAGACACCTGCTATTGGAC |
| ***Smarca4 R*** | GGCTACTTCATACCCTGGGTTC |
| ***Smarca5 F*** | TGATCATGGATCACCTGGAA |
| ***Smarca5 R*** | GCGAACAGCTCTGTCTGCTT |
| ***Promoter 1 F*** | CTGCTATTGGTCTAGGTCTGTG |
| ***Promoter 1 R*** | GATCGACATGAACTCTGGTCTT |
| ***Promoter 2 F*** | GGTATGGAGGTCAGTACCTAGAA |
| ***Promoter 2 R*** | GGCATTCCCAAGATCGACAT |
| ***Promoter 3 F*** | TGTTGGAAGCAGAGGGTATG |
| ***Promoter 3 R*** | GAACTCTGGTCTTGTCCCTAC |
| ***CTCF 1 F*** | GGGAGAAGGATGCATAGACC |
| ***CTCF 1 R*** | CAGGGCGTGCGTAAGGT |
| ***CTCF 2 F*** | TCCTAGGTTCTGTTGCTCTC |
| ***CTCF 2 R*** | CAATGTAAATGTGTGTTTGTGC |
| ***CTCF 3 F*** | TCTGACTGTACACACAGTTAAAGAA |
| ***CTCF 3 R*** | CACCCATGGAGGTCTGATTAC |
| ***DHS 1 F*** | GGATACCGCAGCTAGGAATAAT |
| ***DHS 1 R*** | CGGTCCAAGAATTTCACCTCTA |
| ***DHS 2 F*** | CCGCAGCTAGGAATAATGGAATAG |
| ***DHS 2 R*** | TAGCGGCGCAATACGAATG |
| ***DHS 3 F*** | CGGAACTGAGGCCATGATTAAG |
| ***DHS 3 R*** | GCGCCGGTCCAAGAATTT |

**Supplementary Table 1. Primer sequences used in this study.**

**References (Supplementary information)**

1. Blumli S, Wiechens N, Wu MY, Singh V, Gierlinski M, Schweikert G, Gilbert N, Naughton C, Sundaramoorthy R, Varghese J *et al*: **Acute depletion of the ARID1A subunit of SWI/SNF complexes reveals distinct pathways for activation and repression of transcription**. *Cell Rep* 2021, **37**(5):109943.

2. Chen X, Xu H, Yuan P, Fang F, Huss M, Vega VB, Wong E, Orlov YL, Zhang W, Jiang J *et al*: **Integration of external signaling pathways with the core transcriptional network in embryonic stem cells**. *Cell* 2008, **133**(6):1106-1117.

3. Davis CA, Hitz BC, Sloan CA, Chan ET, Davidson JM, Gabdank I, Hilton JA, Jain K, Baymuradov UK, Narayanan AK *et al*: **The Encyclopedia of DNA elements (ENCODE): data portal update**. *Nucleic Acids Res* 2018, **46**(D1):D794-D801.

4. **Picard Tools, Broad Institute** [<http://broadinstitute.github.io/picard/>]

5. Kato D, Osakabe A, Arimura Y, Mizukami Y, Horikoshi N, Saikusa K, Akashi S, Nishimura Y, Park SY, Nogami J *et al*: **Crystal structure of the overlapping dinucleosome composed of hexasome and octasome**. *Science* 2017, **356**(6334):205-208.

6. Klein DC, Lardo SM, Hainer SJ: **FACT maintains pluripotency factor expression through gene-distal regulation in embryonic stem cells**. *BioRxiv* 2021.

7. Hodges HC, Stanton BZ, Cermakova K, Chang CY, Miller EL, Kirkland JG, Ku WL, Veverka V, Zhao K, Crabtree GR: **Dominant-negative SMARCA4 mutants alter the accessibility landscape of tissue-unrestricted enhancers**. *Nat Struct Mol Biol* 2018, **25**(1):61-72.

8. Marson A, Levine SS, Cole MF, Frampton GM, Brambrink T, Johnstone S, Guenther MG, Johnston WK, Wernig M, Newman J *et al*: **Connecting microRNA genes to the core transcriptional regulatory circuitry of embryonic stem cells**. *Cell* 2008, **134**(3):521-533.

9. de Dieuleveult M, Yen K, Hmitou I, Depaux A, Boussouar F, Bou Dargham D, Jounier S, Humbertclaude H, Ribierre F, Baulard C *et al*: **Genome-wide nucleosome specificity and function of chromatin remodellers in ES cells**. *Nature* 2016, **530**(7588):113-116.

10. Song Y, Liang Z, Zhang J, Hu G, Wang J, Li Y, Guo R, Dong X, Babarinde IA, Ping W *et al*: **CTCF functions as an insulator for somatic genes and a chromatin remodeler for pluripotency genes during reprogramming**. *Cell Rep* 2022, **39**(1):110626.
